# Improved Method for the Detection and Quantification of *Naegleria fowleri* in Water and Sediment Using Immunomagnetic Separation and Real-Time PCR

**DOI:** 10.1155/2013/608367

**Published:** 2013-10-21

**Authors:** Bonnie J. Mull, Jothikumar Narayanan, Vincent R. Hill

**Affiliations:** Division of Foodborne, Waterborne and Environmental Diseases, Centers for Disease Control and Prevention, National Center for Emerging and Zoonotic Infectious Diseases, 1600 Clifton Road, NE, Mail Stop D-66, Atlanta, GA 30329-4018, USA

## Abstract

Primary amebic meningoencephalitis (PAM) is a rare and typically fatal infection caused by the thermophilic free-living ameba, *Naegleria fowleri*. In 2010, the first confirmed case of PAM acquired in Minnesota highlighted the need for improved detection and quantification methods in order to study the changing ecology of *N. fowleri* and to evaluate potential risk factors for increased exposure. An immunomagnetic separation (IMS) procedure and real-time PCR TaqMan assay were developed to recover and quantify *N. fowleri* in water and sediment samples. When one liter of lake water was seeded with *N. fowleri* strain CDC:V212, the method had an average recovery of 46% and detection limit of 14 amebas per liter of water. The method was then applied to sediment and water samples with unknown *N. fowleri* concentrations, resulting in positive direct detections by real-time PCR in 3 out of 16 samples and confirmation of *N. fowleri* culture in 6 of 16 samples. This study has resulted in a new method for detection and quantification of *N. fowleri* in water and sediment that should be a useful tool to facilitate studies of the physical, chemical, and biological factors associated with the presence and dynamics of *N. fowleri* in environmental systems.

## 1. Introduction

*Naegleria fowleri,* a thermophilic free-living ameba found in freshwater environments, causes primary amoebic meningoencephalitis (PAM), a rare and typically fatal disease in children and young adults [1, 2]. In the USA, *N. fowleri* is commonly detected in warm freshwater environments such as lakes, rivers, inadequately disinfected swimming pools, geothermal waters (e.g., hot springs), thermally impacted surface water (e.g., from power plants), and water distribution systems [3–15]. While *N. fowleri* is generally considered to be widespread in the environment, especially in warm weather geographic areas, environmental factors are likely associated with the distribution of PAM in the USA. In recent years there has been an increase in the geographical distribution of PAM cases in the USA and in 2010, the first confirmed case of PAM was identified in Minnesota, the northernmost USA state in which this infection has been documented [9, 16]. These developments highlight the need for improved environmental detection and quantification methods in order to study the ecology of *N. fowleri* and to evaluate potential risk factors for increased exposure to this ameba. The most probable number (MPN) method is reliant on culture methods developed in the 1970s [17] and consists of spreading *Escherichia coli* over agar plates and observing amebic growth. The method cultures different volumes (usually serial dilutions) in 3–5 replicates each, consuming a lot of supplies and analyst time for reading all the plates, as well as requiring very specialized expertise. Since most species of *Naegleria* are morphologically indistinguishable, molecular methods are becoming rapidly more common as a means of detecting its presence [7, 10, 18–20]. 

In particular, quantitative polymerase chain reaction (qPCR) enables the direct detection and quantification of microorganisms in environmental samples without the need for culture isolation. Previous studies have described qPCR methodologies for the quantification of *N. fowleri* in water solely using cultured amebas under laboratory controlled conditions [21, 22]. In one study, the method was applied to a small sample set (*n* = 6) of drinking water distribution biofilms and water [20], and only one study has reported application of qPCR to naturally occurring *N. fowleri* in surface water [19]. Environmental application data are important because qPCR is susceptible to inhibitors found in water samples, particularly concentrated water samples.

Immunomagnetic separation (IMS) has been widely and successfully used to remove inhibitors and concentrate a variety of other microbes from water samples, but only one study has reported its use on *N. fowleri* [23]. The attachment of *N. fowleri*-specific antibodies [24] to the surface of the beads allows capture and isolation of intact amebas directly from a complex matrix in a volume suitable for subsequent detection and enumeration. 

The overall objectives of the present *N. fowleri* study were to (1) develop and evaluate the sensitivity and specificity of an IMS method, (2) develop a novel qPCR assay that can be used to quantify *N. fowleri* and (3) evaluate the optimized method with laboratory controlled seeded experiments and environmental samples. 

## 2. Methods

### 2.1. Ameba Preparation

Four genotypes of *N. fowleri*, identified as CDC:V212, CAMP, CDC:V515, and ATCC 30462, were grown axenically at 42°C in modified Nelson's medium (Schuster 2002 [25]).

*Hartmannella vermiformis* ATCC 50237, *Naegleria australiensis* ATCC 30958, *Naegleria dunnebackei* ATCC PRA-166, and *Naegleria jadini* ATCC 30900 were grown axenically at 37°C in modified PYNFH medium (ATCC 1034). After growth medium was removed, trophozoites were harvested in 1 mL of WB saline [26] and gently dislodged using a cell scraper. *Echinamoeba exundans* ATCC 50171, *Naegleria clarki* ATCC 30544, *Naegleria gruberi* ATCC 30877, *Naegleria italica* ATCC PRA-153, *Naegleria lovaniensis* ATCC 30811, *Naegleria lovaniensis* ATCC 30467, *Tetramitus jugosus* ATCC 30703, *Vahlkampfia inornata* ATCC 30965, and *Vahlkampfia lobospinosa* ATCC 30298 were grown at 37°C on nonnutrient agar (NNA) plates spread with *Escherichia coli* (ATCC 11775). Trophozoites or cysts were harvested in 1 mL of WB saline by gently scraping the surface of the agar plate with a cell scraper. Trophozoites were harvested 1-2 days after passing the culture to a new flask or plate, whereas cultures were left on NNA plates for 14 days to allow for all amebas to form cysts. To create a mixture of trophozoites and cysts, cultures were harvested after 4-5 days. All ameba stock concentrations were determined by four counts on a Thoma hemocytometer using 400x total magnification on a standard light microscope. After an appropriate dilution, this suspension was added to the water sample to obtain the desired seed level for all experiments. 

### 2.2. Water Samples

IMS recovery experiments were performed with both amended deionized (DI) water and Georgia (GA) lake water. Deionized water was amended with 0.01 M phosphate buffered saline (PBS) to produce water samples having a conductivity of 100 *μ*S/cm. To ensure there were no amebas in the lake water used for experiments, the water was filtered through 2.7 *μ*m cellulose acetate filter. IMS specificity experiments were only conducted in the filtered lake water. Unfiltered GA lake water was used in the whole method comparison experiments; therefore a negative nonseeded control sample was taken through the whole procedure to ensure there was no *N. fowleri* present prior to seeding.

### 2.3. Immunomagnetic Separation (IMS) Procedure

Dynabeads Biotin Binder (Invitrogen number 110.47) was precoated with Biotin-labeled anti-*N. fowleri* monoclonal Antibody Nf-5D12 [27] (Indicia Biotechnology, Oullins, France) at a concentration of 2 *μ*g of biotinylated antibodies per 50 *μ*L of Dynabeads and used within 2 weeks. The mixture was allowed to incubate for 60 min with gentle rotation and then placed on the magnet for 2 min and washed with buffer containing 0.01 M phosphate buffered saline pH 7.4, 0.1% bovine serum albumin, and 2 mM EDTA, to remove any unbound antibodies. To each water sample not exceeding 10 mL in volume, 50 *μ*L of freshly vortexed bead-antibody mixture was added and allowed to incubate on the rotator for 30 min. A Leighton tube was placed on the Dynal MPC-6 magnet for 3 min; after the supernatant was discarded the bead pellet was resuspended in 1 mL of buffer, and the separated particles then transferred to a 1.5 mL centrifuge tube. The tube was placed on the Dynal MPC-S magnet for 2 min, supernatant was discarded again, and finally bead pellet was resuspended by vortexing in 100 *μ*L of buffer.

### 2.4. IMS Recovery and Specificity Experiments

The four genotypes of *N. fowleri* used in this study were seeded individually in both cyst and trophozoite forms at a level of 1 × 10^5^ amebas in 5 mL of amended DI water and filtered lake water. The seeded samples were then subjected to IMS in 6 replicate experiments. In addition, a mixture of 1 × 10^5^ cyst (53 ± 18%) and trophozoite (47 ± 18%) forms of 8 *Naegleria* spp., and 5 non-*Naegleria* amebas was seeded separately into 5 mL of filtered surface water in 3 replicate experiments. After the IMS procedure, the recovered amebas were counted microscopically using the hemocytometer as previously described to determine recovery efficiency of the method. One-way analysis of variance was used to test for significant differences between the mean recovery efficiencies and ameba species, ameba stage, ameba genotype, and water type using JMP v10. An alpha value of 0.05 was used for all statistical tests.

### 2.5. Nucleic Acid Extraction, Real-Time PCR Assay Design and Conditions

Nucleic acid extraction was performed on half of the IMS concentrate and the entire harvested cultured sample by a previously reported procedure using a noncommercial lysis buffer containing 4.5 M guanidinium isothiocyanate and bead beating using acid washed zirconium oxide beads [28]. 

The following TaqMan assay primers and probe sequences were selected for testing: (JBVF, 5′-AGG TAC TTA CGT TAG AGT GCT AGT-3′), (JBVR, 5′-ATG GGA CAA TCC GGT TTT CTC A-3′) and the (FAM-) labeled probe (JBVP, 5′-FAM-AC GCC CTA GCT GGT TAT GCC GGA TT-BHQ1-3′) and were evaluated for melting temperature (*T*_*m*_), secondary structure and complementarity. The relative positions of the forward primer, reverse primer and probe are 166–189, 269–290 and 239–263 respectively based on GenBank accession number AJ132020. The primers amplify a 123 bp segment of the ITS region. Reactions were carried out in a 50 *μ*L final reaction mixture using the QuantiTect probe PCR kit (Qiagen, Carlsbad, CA, USA), 5 *μ*L of template DNA, 250 nM of the forward and reverse oligonucleotide primers, 100 nM of FAM-labeled probe, 1.0 *μ*L of 50x nonacetylated BSA (Sigma-Aldrich St. Louis, MO, USA), and 2.5 *μ*L of 20x T4 Gene 32 Protein (New England Biolabs, Ipswitch, MA). In each experiment, *N. fowleri* CDC:V212 DNA transcripts were included as a positive control and PCR-grade water was used as a negative control. All samples and dilutions were tested in duplicate on an ABI 7500 Real-time PCR Detection System (Life Technologies Corp, Carlsbad, CA, USA). Cycling conditions were (i) 95°C for 15 min (activation of *Taq* DNA polymerase) and (ii) 45 cycles of 95°C for 15 seconds and 63°C for 33 seconds. Fluorescence was measured at the end of each PCR cycle. A sample was considered positive when duplicate tests resulted in an average cycle threshold (CT) value <41.0. The four genotypes of *N. fowleri*, 8 *Naegleria* spp., and 5 non-*Naegleria* amebas previously described were tested in triplicate to determine specificity of the TaqMan assay. The DNA amount from each isolate culture used in specificity testing was equivalent to 1000 amebas/reaction; determined by hemocytometer count. A standard curve for the real-time PCR assay was tested in triplicate and prepared using a tenfold dilution series of DNA extracted from a hemocytometer-titered stock of *N. fowleri* CDC:V212 in DI water.

### 2.6. Whole Method Comparison Experiments

A cyst (38 ± 20%) and trophozoite (62 ± 20%) mixture of *N. fowleri* (CDC: V212) was added to 1 L of unaltered surface water samples in four replicate experiments. Samples were processed in parallel procedures, one with the IMS procedure and one without (the control). Each 1 L sample was concentrated by centrifuging two, 500 mL volumes in a Jouan 415 centrifuge at 1,500 × g for 15 min. After centrifugation, the supernatant was carefully removed, the pellet was resuspended, and the centrifuge tube was rinsed with WB saline. For the experimental samples, the pellet was further concentrated using the IMS procedure and the final 100 *μ*L volume was split into three aliquots: 40 *μ*L for agar culture, 40 *μ*L for DNA extraction and real-time qPCR, and 20 *μ*L for direct counting on a microscope. The control samples pellets were also split into aliquots: 1 mL for agar culture and 750 *μ*L for DNA extraction and real-time qPCR. Agar culture plates were spread with *E. coli* prior to sample addition and then incubated for 7 days at 42°C. The entire culture plate was then harvested in 750 *μ*L of WB saline by gently scraping the surface with a cell scraper, and the DNA was extracted before assay by real-time PCR.

### 2.7. Method Evaluation Detecting Naturally Occurring *N. fowleri* in Lake Water and Sediment Samples

**Figure 1:**
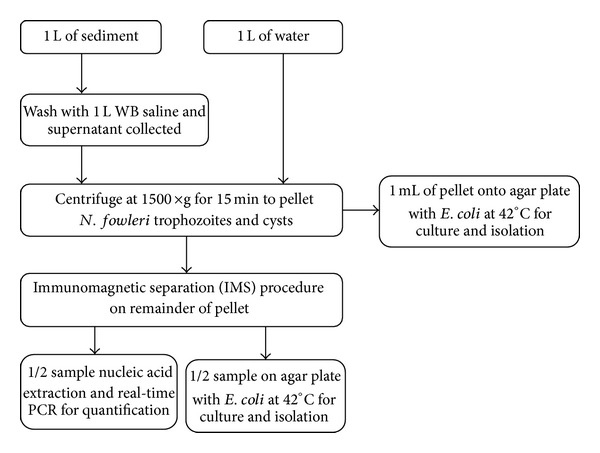
Sediment and water sample processing procedure.

In August and October 2011, 1 L water and sediment samples were collected from 10 lakes in Minnesota and 6 lakes in Florida. Each water and sediment sample was a composite from 4 sites along a beach area at each lake. Sediment samples were washed twice with 500 mL of WB saline and the supernatant was processed using the same procedure as the water samples (Figure 1). Each sample was concentrated by centrifugation as already described, then 1 mL of the resulting pellet was placed on an agar culture plate and the remaining pellet volume was processed using the IMS procedure. The resulting IMS concentrate was split in half: 50 *μ*L was added to an agar culture plate and 50 *μ*L was extracted and assayed using real-time qPCR. 

## 3. Results

### 3.1. IMS Recovery and Specificity Experiments

Overall, not separated by ameba stage or water type, the IMS method was determined to recover 75 ± 17.7% of *N. fowleri* amebas (trophozoites and cysts separately) seeded in 5 mL water samples. As shown in Table 1, *N. fowleri* genotypes were more effectively recovered in trophozoite form than in cyst form. Average recoveries of *N. fowleri* were significantly higher for trophozoites (88 ± 8.4%) than cysts (61 ± 13.2%) when recovery data were analyzed without stratification for genotype or water type (*P* < 0.0001). No significant differences in recoveries were found between genotypes (*P* = 0.5741) or between the amended DI water and lake water (*P* = 0.4046).

The IMS method exhibited little binding to nonpathogenic *Naegleria* species and other free-living ameba. As shown in Table 2, out of the thirteen nontarget amebas tested, only *Vahlkampfia lobospinosa, Naegleria lovaniensis, Naegleria italica,* and *Naegleria clarki* showed a marginal degree (higher than 5% recovery) of cross-reactivity. The cross-reactivity efficiencies observed for non-*N. fowleri* amebas were significantly lower than these deserved for *N. fowleri* (*P* < 0.0001).

### 3.2. Real-Time PCR Assay Development

**Figure 2:**
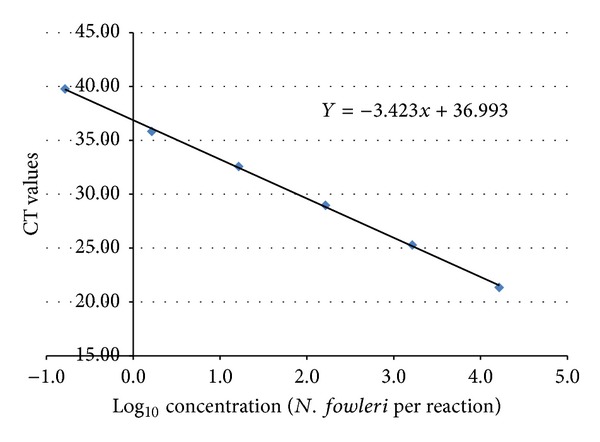
Standard curve for newly designed *N. fowleri* real-time PCR assay using strain CDC:V212 DNA extract.

The qPCR assay detected all 4 genotypes of *N. fowleri* and when tested against the specificity panel of other *Naegleria* spp., and non-*Naegleria* amebas it did cross-react with one non-*Naegleria* ameba. The assay reported a false positive average CT values of 39.2 ± 1.0 for *Hartmannella vermiformis* (data not shown). The assay resulted in average CT values of 28.0 ± 3.1 for the 4 genotypes of *N. fowleri* (data not shown). Using triplicate tenfold dilutions of the DNA stock, a standard curve for the *N. fowleri* PCR TaqMan assay was developed and found to be linear over a range from 10^0^ to 10^4^ amebas per reaction (Figure 2). This testing indicated that the assay could be used to detect as low as 1 ameba per reaction. Using the formula, *E* = 10^(−1/slope)^ − 1, the calculated PCR efficiency for this assay was 96%. 

### 3.3. Whole Method Comparison Experiments

The limit of detection for the sample processing method in conjunction with qPCR was determined, both for direct PCR without culture and PCR following culture. In these experiments, a concentration of 63 amebas/L could be consistently detected (3 of 4 samples positive) when IMS was used to process the samples and qPCR used for direct analysis (i.e., without prior culture) (Table 3). When IMS was not used, the direct PCR method detection limit appeared to be slightly higher. Similarly, IMS appeared to enable a lower method detection limit when culture of the pelleted samples was performed prior to PCR. Consistent PCR detection after culture was observed for a seed level of 388 amebas/L when IMS was used (4 of 4 samples positive) but was higher than 388 amebas/L when IMS was not used. The use of IMS also appeared to result in the *N. fowleri* cultures being capable of more easily replicating based on the substantially lower CT values associated with post-IMS cultures. 

### 3.4. Detection of Naturally Present *N. fowleri* in Lake Water and Sediment Samples

When the *N. fowleri* testing procedure was used to analyze 16 natural (nonseeded) lake water and 16 sediment samples, *N. fowleri* was detected by PCR in 6 sediment samples and 4 water samples (Table 4). Postculture detection appeared to be improved when IMS was used to process sediment samples (6 detections following IMS versus 2 detections when IMS was not used). However, the only two water samples that were positive for *N. fowleri* after culture were from the procedure when IMS was not used. Direct PCR was effective for rapidly detecting *N. fowleri* in only 3 of the 10 samples (water and sediment) that were found to be positive for *N. fowleri* but was the only technique that enabled detection of *N. fowleri* in two water samples (FL5 and FL6). 

### 3.5. Quantification Estimates of *N. fowleri* in Lake Water and Sediment Samples

**Figure 3:**
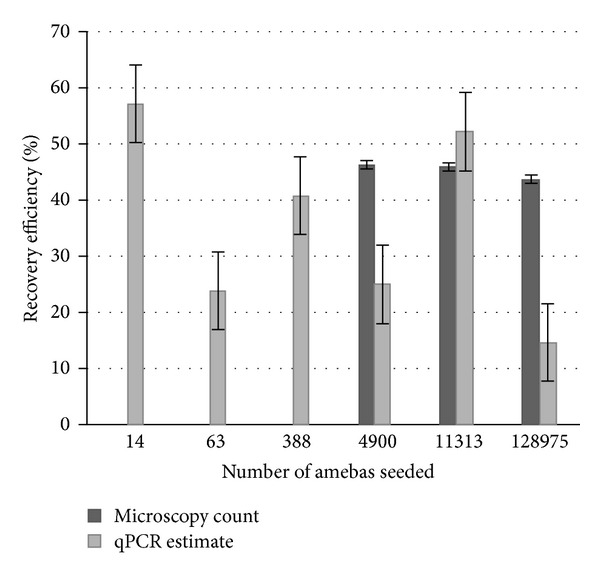
Recovery efficiency of *N. fowleri* in 1 L of seeded GA lake water.

Using CT values from seeded and nonseeded sample testing, estimates of *N. fowleri* concentrations were made and compared to known concentrations when possible. Using this method, qPCR estimates were within an order of magnitude of the known amount of amebas in samples determined by microscopy (Table 5). Using the qPCR concentration estimates for the seeded samples, the sample processing method (including IMS) had an average recovery of 36 ± 16%. The 3 highest seed levels were also able to be directly counted using the hemocytometer and had an average recovery of 45 ± 5%.  Figure 3 shows that the recovery efficiencies estimated using qPCR results were on average lower and more variable than the recoveries determined by direct microscopy counts.

## 4. Discussion

The results of this study demonstrate that the reported sample processing and analytical procedure can reliably detect and quantify *N. fowleri* in sediment and water samples. This method builds upon the standard method of centrifugation and culture with *E. coli* at 42°C, with the addition of IMS to remove inhibitors and competing microorganisms and real-time PCR to enable quantification without having to employ the laborious MPN technique. The real-time PCR assay reported in this study was determined to be able to detect as low as 1 ameba per reaction, likely because it targets the 18S gene that has multiple copies in each ameba. The specificity testing revealed that both the qPCR assay and IMS method are not 100% specific. However, even with the mild cross-reactivity that was observed, by combining the two methods the overall water and sediment testing procedure should result in specific detections of *N. fowleri*. 

IMS has been used with varying effectiveness to recover a variety of microorganisms from environmental samples. The overall recovery and range of 75 ± 17.7% *N. fowleri* amebas for the IMS method in Table 1 is comparable to that seen with protozoan parasite IMS methods. *Cryptosporidium* spp. IMS recovery has been reported to range from 4.8% to 147.4% in water with turbidities up to 615 NTU [29]. Similarly, *Giardia* spp. cyst IMS recovery efficiencies have been reported to range from 20 to 81%, decreasing proportionally as turbidity increased [30]. The greater number of positive detections reported in the present study for both seeded experiments and naturally present samples shows the benefit of using IMS to further concentrate the sample before culturing. Culturing *N. fowleri* from environmental samples can lack detection sensitivity due to overgrowth from more rapidly growing but nonpathogenic *Naegleria* species such as *N. lovaniensis* and other free-living amebas [31]. Since the IMS method was found to only minimally cross-react with nonpathogenic amebas, these nontarget amebas should be removed during the procedure. Therefore, with fewer competing microorganisms being present, *N. fowleri* may proliferate more readily during culture and exhibit less inhibition during qPCR, resulting in the lower CT values and the greater number of detections reported in Table 3. For direct real-time PCR analysis, it appears that the limit of detection for the *N. fowleri* testing method developed in this study is on the order of 10–100 amebas/L (Table 5). The three samples where naturally occurring *N. fowleri* were directly detected in the sample and estimated to be from 2.7 to 15 amebas/L indicates that low levels of *N. fowleri* can be detected in natural water and sediment samples. Other studies to date that have quantified naturally occurring *N. fowleri* by qPCR found a range from 7 to 256 copies/100 mL in a Texas lake and <1 to >86 cells/250 mL in Australian distribution system drinking water [19, 20]. These data provide further evidence that it is important for an environmental sample testing method for *N. fowleri* to have a low limit of detection. It has been shown in numerous studies that *N. fowleri* can persist at low levels in lake water, especially during colder months when it is thought that the amebas “overwinter” in sediment [8, 14, 27, 32, 33]. When temperatures increase to 30–40°C, *N. fowleri* concentrations have been shown to increase, with associated increased frequency of detections [32, 34, 35]. 

When the *N. fowleri* testing protocol developed in this study was applied to nonseeded water samples (Table 4), the results indicated that direct PCR could be effective for detecting *N. fowleri* (and thereby enabling estimation of *N. fowleri* concentration by qPCR) but culture (with or without IMS) resulted in additional *N. fowleri* detections. It is not clear whether this was due to the sample volume tested (relatively lower for PCR than for culture), PCR inhibition, or other factors. However, the results indicate that the overall sampling analysis protocol for *N. fowleri* in environmental systems should be comprehensive in order to effectively detect and quantify *N. fowleri*. Real-time PCR can play a role for direct analysis and rapid screening of samples from a study site, but performing culture procedures for *N. fowleri* (with and without prior IMS) would be prudent.

The IMS and qPCR methods reported in this study should be valuable tools to facilitate ecological studies of *N. fowleri* in water systems in which detection and quantification data are needed for comparison of optimal and nonoptimal growth conditions. The need for such research has been recently highlighted for determining the engineering and ecologic factors contributing to higher densities or diversity of *N. fowleri* [36] and potentially using *N. fowleri* concentrations in surface water and drinking water distribution systems as a complementary health risk indicator [15].

## Figures and Tables

**Table 1:** IMS recovery efficiency for *N. fowleri* seeded into 5 mL of water (*n* = 6).

Genotype (strain)	Water type	Cyst stage (average ± SD)	Trophozoite stage (average ± SD)
I (CDC:V212)	Amended DI	50 ± 11%	83 ± 9%
	Lake*	67 ± 7.9%	85 ± 5.5%
II (CAMP)	Amended DI	71 ± 7.6%	77 ± 2.5%
	Lake	68 ± 2.8%	82 ± 10%
III (CDC:V515)	Amended DI	62 ± 13%	87 ± 6.6%
	Lake	56 ± 14%	94 ± 4.0%
IV (ATCC 30462)	Amended DI	60 ± 21%	97 ± 5.3%
	Lake	65 ± 7.2%	92 ± 6.0%
All 4 genotypes	Amended DI	69 ± 16%	87 ± 9.5%
	Lake	63 ± 10%	89 ± 7.2%

*GA lake water filtered through a 2.7 *μ*m cellulose acetate filter.

**Table 2:** IMS recovery efficiency for non-*N. fowleri* amebas in 5 mL of lake water (*n* = 3).

ATCC no.	Species	Average ± SD%
30467	*Naegleria lovaniensis *	1.5 ± 1.1%
30811	*Naegleria lovaniensis *	14 ± 3.5%
PRA-166	*Naegleria dunnebackei *	2.8 ± 0.6%
PRA-153	*Naegleria italica *	9.0 ± 2.8%
30958	*Naegleria australiensis *	2.7 ± 2.5%
30544	*Naegleria clarki *	8.5 ± 3.4%
30877	*Naegleria gruberi *	3.4 ± 0.7%
30900	*Naegleria jadini *	1.5 ± 0.6%
30965	*Vahlkampfia inornata *	1.3 ± 0.7%
30298	*Vahlkampfia lobospinosa *	14 ± 3.3%
30703	*Tetramitus jugosus *	0.6 ± 0.3%
50171	*Echinamoeba exundans *	3.9 ± 0.9%
50237	*Hartmannella vermiformis *	1.0 ± 0.2%

**Table 3:** *N. fowleri *detection in seeded 1 L lake water samples.

Seed level	Direct PCR without culture				Culture followed by PCR confirmation			
	IMS		No IMS		IMS		No IMS	
	CT	Pos^†^ no.	CT	# Pos	CT	Pos no.	CT	# Pos
14	39.1	1	38.0	1	Negative	0	38.9	1
63	38.5	3	40.5	2	32.8	1	34.6	2
388	35.1	4	36.7	4	27.8	4	38.3	1
4,900	31.9	4	33.2	4	28.9	4	35.7	4
11,300	29.83	4	29.96	4	23.99	4	34.87	4
129,000	27.87	4	28.19	4	25.38	4	31.57	4

^†^Number of positive results out of four replicate tests (all conditions were repeated four times).

**Table 4:** Detection by PCR of naturally present *N. fowleri *in lake water and sediment samples.

Sample ID	Direct		Culture: after IMS		Culture: no IMS	
	Sediment	Water	Sediment	Water	Sediment	Water
MN1	−	−	+	−	−	+
MN2	−	−	−	−	−	−
MN3	−	−	−	−	−	−
MN4	−	−	+	−	−	−
MN5	−	−	−	−	−	+
MN6	−	−	−	−	−	−
MN7	−	−	−	−	−	−
MN8	−	−	+	−	−	−
MN9	+	−	+	−	−	−
MN10	−	−	−	−	−	−
FL1	−	−	−	−	−	−
FL2	−	−	−	−	−	−
FL3	−	−	−	−	−	−
FL4	−	−	−	−	−	−
FL5	−	+	+	−	+	−
FL6	−	+	+	−	+	−

**Table 5:** Estimated concentration of *N. fowleri* in water and sediment samples.

Sample ID	No. of amebas seeded	Average CT value	Estimated concentration (amebas/L)
GA water	14	39.1	8
GA water	63	38.5	15
GA water	388	35.1	183
GA water	4,900	31.9	1,224
GA water	11,300	29.8	5,902
GA water	129,000	27.9	18,620
FL5 water	NA	40.6	2.7
FL6 water	NA	38.2	15
MN9 sediment	NA	38.3	13
WB saline control	9,800	28.8	7,900
WB saline control	37,000	27.8	15,000
